# Prevalence and penetrance of pathogenic and likely pathogenic *LDLR* and *APOB* gene variants linked to familial hypercholesterolemia and increased risk of ischemic heart disease

**DOI:** 10.3389/fgene.2025.1589014

**Published:** 2025-08-25

**Authors:** I. K. Dzhumaniiazova, A. N. Meshkov, V.V. Daniel, M. V. Ezhov, E. A. Zelenova, U. V. Chubykina, D.A. Kashtanova, M. V. Ivanov, L. R. Matkava, O. I. Blinova, N. A. Kumar, A. Y. Fedorov, H. U. Ibragimova, T. A. Lavrikova, Y. O. Aksenova, T. M. Gurciev, N. V. Gomyranova, Y. S. Vorobeva, Z. B. Hasanova, V. S. Yudin, V. V. Makarov, A. A. Keskinov, S. A. Kraevoy, S. A. Boytsov, S. M. Yudin, V. I. Skvortsova

**Affiliations:** ^1^ Federal State Budgetary Institution «Centre for Strategic Planning and Management of Biomedical Health Risks» of the Federal Medical and Biological Agency, Moscow, Russia; ^2^ Federal State Budgetary Institution National Medical Research Centre of Cardiology Named After Academician E.I. Chazov of the Ministry of Health of the Russian Federation, Moscow, Russia; ^3^ Federal State Budgetary Institution National Medical Research Center for Therapy and Preventive Medicine of the Ministry of Healthсare of the Russian Federation, Moscow, Russia; ^4^ Federal Medical and Biologicl Agency, Moscow, Russia

**Keywords:** *low-density lipoprotein receptor*, *apolipoprotein B*, familial hypercholesterolemia, whole-genome sequencing, Russia

## Abstract

**Background:**

Familial hypercholesterolemia (FH) is a prevalent hereditary disorder, with its monogenic form linked to an elevated risk of early-onset ischemic heart disease. Evaluating the prevalence and penetrance of pathogenic and likely pathogenic variants associated with this disorder would provide valuable information supporting routine FH screening of the general population. Such informed screening would facilitate early identification of at-risk individuals, enabling timely intervention and management.

**Methods:**

We analyzed genetic data from 4,856 individuals with various cardiovascular conditions for pathogenic and likely pathogenic variants in the PCSK9, APOB, and LDLR genes. The evaluation included comprehensive clinical assessments, instrumental examinations, and laboratory tests. All genetic data were obtained through the whole-genome sequencing of blood leukocytes.

**Results:**

A total of 1.77% of participants carried pathogenic or likely pathogenic variants in the LDLR or APOB genes, and none in the PCSK9 gene. After adjusting for sex and age, the risk of ischemic heart disease was 1.3 times higher in carriers of pathogenic or likely pathogenic variants [95% CI 1.18–1.46; *p* = 5*10-7]. Additionally, the carriers presented with significantly higher levels of total cholesterol and LDL-C (*p* = 0.00032 and *p* = 0.0123, respectively).

**Conclusion:**

FH remains significantly underdiagnosed. Only 10.5% of carriers of pathogenic or likely pathogenic variants in the LDLR and APOB genes had a prior diagnosis of FH. Our findings suggest low diagnostic rates for this disorder in Eastern European populations and highlight the need for routine genetic screening of younger individuals. However, further research is needed to assess the clinical applicability and cost-effectiveness of such screening programs.

## Introduction

Familial hypercholesterolemia (FH) is a monogenic disorder that is primarily inherited in an autosomal-dominant manner. Individuals with FH typically present with elevated levels of total cholesterol and low-density lipoprotein (LDL), which increases their risk of developing cardiovascular diseases (CVDs). Two meta-analyses published in 2020 found that the prevalence of FH varied across geographic regions, ranging from approximately 1 in 311 to 1 in 313 individuals (Hu et al., 2020; Beheshti Sabina et al., 2020). A meta-analysis published in 2023 showed that the prevalence of phenotypically defined FH in children and adolescents in the United States ranged from 0.2% to 0.4% (1:250 to 1:500) (Guirguis-Blake et al., 2023). Genetic screening increases the detected prevalence of FH. For example, whole-genome sequencing (WGS) of over 6,000 newborns in China revealed an FH frequency of 0.47% (95% CI: 0.32%–0.66%) (Zhou et al., 2024). The Epidemiology of Cardiovascular Diseases and Their Risk Factors in Regions of the Russian Federation (ESSE-RF) study estimates that 0.58% of Russians, or approximately 1 in 173 individuals, are diagnosed with either FH or probable FH (Meshkov et al., 2021a).

Monogenic FH and polygenic dyslipidemia (PD) exhibit different clinical courses. Consequently, in addition to a prompt diagnosis, accurately distinguishing the specific type of FH presents an important challenge. Although the LDL levels may be similar in FH and PD patients, individuals with FH face a significantly higher risk of prematurely developing ischemic heart disease (IHD) and tend to be less responsive to lipid-lowering drug therapy (Tandirerung, 2022). Initiating treatment for FH at an early stage significantly reduces the risk of CVDs (Bouhairie and Goldberg, 2015).

The current diagnosis and treatment strategies for FH need substantial improvement. Although genetic testing is a standard method for FH diagnosis, its availability and routine implementation remain limited in many countries, resulting in clinicians relying primarily on the clinical symptoms of the disease. A study involving patients with acute coronary syndrome (ACS) in Japan followed the Japanese Atherosclerosis Society (JAS) guidelines (2017) and found that only 1.9% of the patients satisfied the diagnostic criteria for FH, while none of the FH patients carrying pathogenic FH-associated variants met the criteria (Harada-Shiba et al., 2022).

FH patients predominantly carry mutations in the following genes: the low-density lipoprotein receptor (*LDLR*), apolipoprotein B (*APOB*), and proprotein convertase subtilisin/kexin type 9 (*PCSK9*) (Chora et al., 2022; Di Taranto et al., 2020). The American College of Medical Genetics and Genomics recommends reporting pathogenic and likely pathogenic variants (PVs and LPVs) in three FH-associated genes: *APOB*, *LDLR*, and *PCSK9* (Richards et al., 2015). The penetrance of PVs and LPVs associated with severe FH may range from 25% to 90%. However, carrying a PV or LPV is an independent risk factor for IHD, and its detection may guide IHD-associated prevention and treatment strategies (Meshkov et al., 2021a; Dikilitas et al., 2023; Vaezi and Amini, 2022; Khera et al., 2016).

This study assessed the penetrance and prevalence of PVs and LPVs in the three FH-associated genes—*LDLR*, *APOB*, and *PCSK9*—in a large and comprehensively examined cohort recruited from inpatients at the National Medical Research Center of Cardiology named after academician E. I. Chazov. Additionally, it examined the effects of these on the risk of IHD.

## Methods

### Participants

We examined the medical records of 4,856 inpatients at the National Medical Research Center of Cardiology named after academician E.I. Chazov. These patients participated on an all-comers basis in an ongoing joint research study focused on molecular diagnostic markers of CVDs (ClinicalTrials identifier: NCT06253481; Genetics of Cardiovascular Diseases (GCVD)); this study was conducted collaboratively by the National Medical Research Center of Cardiology named after academician E.I. Chazov and the FMBA’s Centre for Strategic Planning.

### Clinical examination

The participants underwent a comprehensive examination and testing process in accordance with the established clinical guidelines for their principal CVD diagnosis at the time of hospitalization. The examination included clinical and instrumental evaluations, such as electrocardiography, echocardiography, Doppler sonography of the innominate and femoral arteries (if indicated), and coronary angiography (if indicated). Blood samples were collected from all participants for standard laboratory testing, such as a complete blood count, blood chemistry and lipid metabolism, coagulation, highly sensitive troponin T and I (if indicated), and biobanking.

### Ethical considerations

The study was approved by the Ethics Committee of the National Medical Research Center of Cardiology named after academician E.I. Chazov (protocol no. 271, dated 27 September 2021). All patients provided informed consent to participate in the study and have their blood and serum samples collected and stored for future genetic research and biobanking initiatives.

### Whole-genome sequencing

Genomic DNA (gDNA) from whole blood samples was extracted using the MagAttract HMW DNA Kit (QIAGEN, Germany), according to the manufacturer’s protocol. The yield and purity of the isolated gDNA were manually determined using the Infinite F Nano+ Plate Reader (Tecan, Switzerland) and the NanoDrop 8000 Microvolume UV–VIS Spectrophotometer (Thermo Fisher Scientific, United States), respectively. Only gDNA samples with absorbance ratios A260/280 of 1.7–1.9 and A230/260 of 1.8–2.2 were selected for further analysis.

A total of 150–500 ng of gDNA was used to prepare next-generation sequencing (NGS) libraries. Libraries were prepared using the Illumina DNA Prep Kit (Illumina, Inc., United States), according to the manufacturer’s recommendations, using the Tecan Freedom EVO Robotic Station (Tecan, Switzerland). gDNA concentrations in the library samples were measured using the Infinite F Nano Plus Tablet Reader (Tecan, Switzerland). The size of the resulting libraries was determined using the Agilent D1000 Reagent Kit on the Agilent 4200 TapeStation (Agilent Technologies, Inc., United States). Pooling was performed automatically using a Tecan Freedom EVO Robotic Station (Tecan, Switzerland). Each pool was diluted to a final gDNA concentration of 1.5 nM prior to sequencing. Pool quality control was performed using the Agilent High-Sensitivity D1000 Screen Tape Reagent Kit on the Agilent 4200 TapeStation (Agilent Technologies, Inc., United States).

WGS was performed on an Illumina NovaSeq 6000 System (Illumina, Inc., United States) using the S4 reagent kit (Illumina, Inc., United States) for 300 cycles, generating 2 × 150 bp paired-end reads with a minimum coverage of 30 × (>350 million reads). The Illumina DRAGEN Bio-IT Platform (Illumina, United States) was used to align reads to the reference genome (GRCh38). Small variant calling was performed using Strelka2 for small cohorts (Illumina, United States) (Kim et al., 2018).

### Selection of variants potentially associated with FH

We examined variants in the *LDLR*, *APOB*, and *PCSK9* genes, as recommended by the American College of Medical Genetics (ACMG, 2022), for their association with FH (Miller et al., 2022).

• The variants analyzed included single-nucleotide substitutions and indels located within exons or splice sites, with a minor allele frequency (MAF) below 0.01% in gnomAD, and variants not currently reported in gnomAD.
• The variants classified as benign or likely benign according to ClinVar (as of 04/24/2023) were excluded from analysis.

In addition to manual review, we used the ClinVar database and the InterVar automatic interpreter (accessed on 27 July 2021) (Li and Wang, 2017). InterVar assesses variants against 18 out of 28 ACMG criteria (PVS1, PS1, PS4, PM1, PM2, PM4, PM5, PP2, PP3, PP5, BA1, BS1, BS2, BP1, BP3, BP4, BP6, and BP7). This semi-automated approach facilitated the reliable annotation of the extensive number of variants under review. Thus, the final variant annotation was based on integrating InterVar interpretations, ClinVar data, and other available evidence.

The reporting of variants annotated as PVs and LPVs in ClinVar was based on their review status in ClinVar and InterVar interpretations.

• No change in annotation was required for PVs and LPVs with a ClinVar review status of “expert panel” or “multiple submitters,” if also interpreted as PVs or LPVs by InterVar.
• Manual review required in two cases:
• Variants classified by InterVar as variants of uncertain significance (VUSs), despite being annotated as PVs and LPVs in ClinVar.
• Variants with a ClinVar review status of “single submitter,” including those marked as “criteria not provided.” The comprehensive review included InterVar interpretations, available data from clinical cases, *in silico* modeling results, and *in silico* interpretations (SIFT<0.05, PolyPhen-2 HDIV ≥ 0.95, MutationAssessor ≥ 2, M-CAP >0.025, and CADD ≥ 15). The agreement between three or more annotators was considered *in silico* evidence of the LPV status. For inconclusive final interpretations, REVEL > 0.75 and MetaLR > 0.5 were applied. PVs with a ClinVar review status of “single submitter” were annotated as pathogenic only if their classification was supported by clinical test results and an ACMG criteria-based pathogenicity assessment.

The reporting of variants annotated as VUSs in ClinVar or not reported in the database was based on their InterVar interpretations, with only VUSs interpreted as PVs and LPVs being further examined:

• No change in annotation was required forPVs and LPVs with a review status of “expert panel.”
• Manual review was required forVUSs with a status of “single submitter” or “multiple submitters.” The review included InterVar interpretations, available data from clinical cases, *in silico* modeling results, and *in silico* interpretations.

Rare missense variants in LDLR with an MAF below 0.0005 were also analyzed and annotated based on their MAF and the predictions using *in silico* tools, such as MetaLR and MetaSVM (Natarajan et al., 2018). To designate variants that may impact gene function but do not currently meet the established ACMG pathogenicity criteria due to insufficient evidence, an additional classification, i.e., “potentially deleterious variants,” was introduced (hereinafter interchangeably referred to as potentially deleterious variants or variants predicted to be deleterious).

### FH diagnoses, LDLR and APOB variant penetrance

The medical records of all participants carrying the variants under review in the *LDLR* and *APOB* genes were examined for a prior diagnosis of FH. In the absence of such a diagnosis, their health data were assessed for heterozygous FH (HeFH) using the Dutch Lipid Clinic Network Score (DLCNS), a validated clinical tool for FH assessment (Bouhairie and Goldberg, 2015).

The penetrance of the variants in *LDLR* and *APOB* was assessed based on the maximum LDL levels obtained from the participants’ medical records. A maximum threshold of ≥4.0 mmol/L and ≥ 4.9 mmol/L was set for *LDLR* and *APOB* variants, respectively (Di Taranto et al., 2020). For patients receiving cholesterol-lowering therapy, pre-treatment LDL levels were quantified using the LDL–cholesterol correction factor table developed by Haralambos et al. (2015).

### Statistical analysis

Statistical analysis and data visualization were carried out using Python v3.9.12 and its libraries: NumPy (v1.21.5), pandas (v1.4.2), Seaborn (v0.11.2), Matplotlib (v3.5.1), and statsmodels (v.0.13.2). Fisher’s exact test was used for categorical variables, and the Mann–Whitney test was used for quantitative variables. The null hypothesis was rejected at *p* < 0.05.

Odds ratios were calculated using logistic regression in statsmodels v.0.13.2 (Python v.3.9.12). To calculate odds ratios for lipid profiles, age, sex, and the intake of lipid-lowering medications of any class were used as covariates. Two models were used to calculate the odds ratio for IHD. The first model included sex and age as covariates, while the second model additionally included BMI, diabetes, and smoking status.

## Results

### Participants

The study included 4,856 participants (2,091 women; 43%). Supplementary Table S1 provides the clinical characteristics of the study participants. Primary diagnoses (ICD-10 codes) were available for 4,770 participants (98.23%).

### Selection of PVs, LPVs, and VUSs predicted to be deleterious

A total of 44 variants in the *LDLR* and *APOB* genes were selected based on the criteria. There were no carriers of LPVs in the *PCSK9* gene in our cohort.

Supplementary Table S2 provides the complete list of the selected variants. A total of 86 participants carried at least one of these variants. Specifically, 63 patients carried a single *LDLR* variant, and four patients carried two *LDLR* variants, with two carriers previously diagnosed with the homozygous form of FH and one carrier potentially meeting the criteria for heterozygous FH based on the medical data analysis. Fifteen patients were carriers of the p.Arg3527Gln variant in the *APOB* gene, and four patients were carriers of the p.Gln4494del variant in the *APOB* gene. Overall, 1.77% of the participants (86 out of 4,856) carried one or more variants.

Data from patients with two variants, a proband relative diagnosed with FH and hospitalized as a result of family cascade screening, and carriers of the p.Gln4494del variant in the *APOB* gene were excluded from the IHD risk analysis. Four patients carried the p.Gln4494del variant in the *APOB* gene, which was previously found to be associated with FH in a single study (Fernández-Higuero et al., 2015). None of the carriers had been taking lipid-lowering drugs, and their LDL-C levels did not exceed 3.5 mmol/L, suggesting the absence of a link between the p.Gln4494del variant and FH development. This variant was not further analyzed.

**FIGURE 1:**
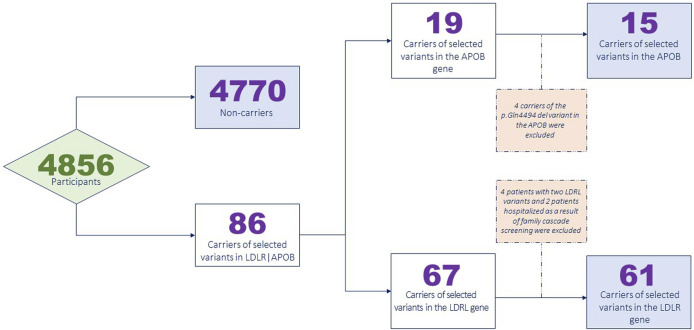
Participant exclusion flowchart.

Figure 1 presents a flowchart illustrating the exclusion of the participants from the analysis.

Supplementary Table S3 shows the final list of variants that were selected to calculate the penetrance, risk of IHD, and dyslipidemia. Overall, 76 participants were found to carry one of the selected variants.

Table 1 presents data on the carriers of PVs, LPs, or VUSs predicted to be deleterious in the *LDLR* and *APOB* genes.

**TABLE 1:** Characteristics of carriers and non-carriers of PVs, LPs, or VUSs predicted to be deleterious in LDLR and APOB.

Parameter	Carriers of PVs, LPs, or VUSs predicted to be deleterious in *LDLR* and *APOB*; n = 76 (N (%) or Me [Q1; Q3])	Non-carries, n = 4,770 (N (%) or Me [Q1; Q3])	Adjusted *p*-value^a^
Men	41 (53.95%)	2,721 (57.04%)	NA
Women	35 (46.05%)	2,049 (42.95%)	NA
Age	65 [56, 73]	66 [58, 74]	0.574
BMI			
Underweight	0 (0%)	38 (0.79%)	0.374
Healthy weight	20 (26.32%)	1,058 (22.18%)	0.421
Overweight (pre-obese)	36 (47.37%)	1,852 (38.84%)	0.103
Obesity I	15 (19.74%)	1,245 (26.11%)	0.212
Obesity II	5 (6.58%)	421 (8.83%)	0.466
Obesity III	0 (0%)	155 (3.25%)	0.1
Smoking			
Non-smoker/former smoker	68 (89.47%)	4,115 (86.27%)	0.424
Nicotine dependence of any degree	8 (10.53%)	655 (13.73%)	0.424
Alcohol consumption (AUDIT-C)			
0 points (never)	43 (56.58%)	2,693 (56.46%)	0.702
1 point (positive: a cumulative score of ≥ 4 in men and ≥ 3 in women)	11 (14.47%)	754 (15.81%)	0.702
Principal diagnosis at discharge			
Hypertensive diseases (I10–I15)	54 (71.05%)	3,492 (73.21%)	0.649
Ischemic heart diseases (I20–I25)	60 (78.95%)	2,551 (53.48%)	5.48^a^10^−7^
Pulmonary heart disease and diseases of pulmonary circulation (I26–I28)	3 (3.95%)	232 (4.86%)	0.561
Pericarditis (I30–I32)	0 (0%)	11 (0.23%)	0.673
Endocarditis and valve disorders (I33–I39)	10 (13.16%)	278 (5.82%)	0.0062
Myocarditis and cardiomyopathy (I40–I43)	1 (1.32%)	177 (3.71%)	0.249
Arrhythmias and conduction disorders (I44–I49)	26 (34.21%)	2,484 (52.08%)	0.00181
Heart failure (I50)	1 (1.32%)	116 (2.43%)	0.542
Other	2 (2.64%)	258 (5.40%)	0.439
Dyslipidemia and hyperlipidemia	23 (30.26%)	1,716 (35.97%)	0.309
Diabetes mellitus	17 (22.34%)	1,049 (21.95%)	1
Lipid and glucose panel			
Cholesterol	4.61 [3.70, 6.18]	4.19 [3.48, 5.14]	0.00031
LDL	2.59 [1.775, 3.975]	2.30 [1.73, 3.11]	0.0117
HDL	1.205 [0.9375, 1.43]	1.17 [0.98, 1.41]	0.6609
Glucose	5.125 [4.8875, 5.8]	5.3 [4.89, 6]	0.704
Triglycerides	1.19 [0.8775, 1.785]	1.32 [0.98, 1.81]	0.2402
Medication			
Antiplatelet drug (acetylsalicylic acid or P2Y12 receptor blockers)	42 (55.26%)	2,360 (49.48%)	0.0870
Oral anticoagulants	16 (21.05%)	1,736 (36.39%)	0.0105
Anti-arrhythmics (beta blockers)	48 (63.16%)	2,963 (62.12%)	0.339
Statins and other lipid-lowering drugs	52 (68.42%)	3,063 (64.21%)	0.0716
Antihypertensive drugs (ACE inhibitors, angiotensin receptor blockers, aldosterone antagonists, and calcium-channel blockers)	45 (59.21%)	3,361 (70.46%)	0.0382

^a^ *p*-value adjusted for sex and age (for age, adjusted for sex only).

^b^ (%): the number of participants in the trait (percentage from the total number of participants in the group), Me [Q1, Q3]: median [quartile 1; quartile 3].

### Clinical profile and IHD risk: a comparison of variant carriers and non-carriers

Table 2 presents the clinical characteristics of selected variant carriers in the *LDLR* and *APOB* genes, whose data were analyzed for the risk of IHD, and the clinical characteristics of the participants who did not carry these variants.

**TABLE 2:** Clinical data for the carriers of the selected variants in *LDLR* and/or *APOB*.

Clinical parameter	Carriers of variants in both *LDLR* and *APOB* (n = 76)	Carriers of *LDLR* variants (n = 61)	Carriers of PVs or LPVs in *LDLR* (based on the ACMG criteria, 2015) (n = 37)	Carriers of VUSs predicted to be deleterious (n = 24)	Carriers of variants in *APOB* (n = 15)
Initial FH diagnosis	8 (10.5%)	8 (13.1%)	8 (21.6%)	0	0
FH diagnosis after medical data analysis	12 (15.2%)	6 (9.8%)	6 (16.2%)	0	6 (40%)
IHD (n and %)	61 (80.3%)	48 (78.7%)	30 (81%)	18 (75%)	13 (86.7%)
Patient’s age at IHD diagnosis	58 [50. 66]	58.5 [50. 66]	54.5 [48. 65]	62.5 [56. 69]	55 [50. 63]
LDL-C level of 4.0 mmol/L or more (n and %)	55 (72.4%)	41 (67.2%)	29 (78.4%)	12 (50%)	14 (93.3%)
LDL-C level of 4.9 or more (n and %)	40 (52.6%)	28 (45.9%)	24 (73%)	4 (16.7%)	12 (80%)

The odds ratio for lipids and the risk of IHD were calculated for the participants in these groups (Tables 3, 4, respectively).

**TABLE 3:** Age-, sex-, and lipid therapy-adjusted odds ratios of lipids for the carriers of PVs, LPVs, or VUSs predicted to be deleterious in *LDLR* and/or *APOB*.

Lipid parameter	Carriers of variants in both *LDLR* and *APOB* (n = 76)	Carriers of *LDLR* variants (n = 61)	Carriers of PVs or LPVs in *LDLR* (based on the ACMG criteria, 2015) (n = 37)	Carriers of VUSs predicted to be deleterious (n = 24)	Carriers of variants in *APOB* (n = 15)
Total cholesterol	1.95 [1.45; 2.61], *p*-value 1.23*10^−05*^	1.93 [1.39; 2.68], *p*-value 9.59*10^−05*^	3.08 [2.03; 4.68], *p*-value 1.66*10^−07*^	0.90 [0.53; 1.53], *p*-value 0.697	1.99 [1.03; 3.84] 0.04^*^
HDL	0.99 [0.91; 1.08], *p*-value 0.92	0.98 [0.89; 1.08], *p*-value 0.847	0.97 [0.86; 1.09], *p*-value 0.817	0.99 [0.86; 1.15], *p*-value 0.912	1.03 [0.87; 1.23] 0.876
LDL	1.63 [1.18; 2.24], *p*-value 00,028^*^	1.53 [1.07; 2.18], *p*-value 0.0196^*^	2.49 [1.55; 3.99], *p*-value 0.0002^*^	0.81 [0.47; 1.39], p*-*value 0.445	2.05 [1.01; 4.16] 0.047^*^
Triglycerides	0.85 [0.66; 1.10], *p*-value 0.277	0.91 [0.69; 1.21], *p*-value 0.528	0.79 [0.55; 1.14], *p*-value 0.0003^*^	1.14 [0.72; 1.79], *p*-value 0.268	0.66 [0.37; 1.15] 0.181

**TABLE 4:** Age; sex only; and age-, sex-, and other cofactors (smoking, diabetes mellitus, and BMI)-adjusted odds ratios of IHD for the carriers of one PV, LPV, or VUS predicted to be deleterious in *LDLR* and/or *APOB*.

IHD	Carriers of variants in both *LDLR* and *APOB* (n = 76)	Carriers of *LDLR* variants (n = 61)	Carriers of PVs or LPVs in *LDLR* (based on the ACMG criteria, 2015) (n = 37)	Carriers of VUSs predicted to be deleterious (n = 24)	Carriers of variants in *APOB* (n = 15)
IHD (age- and sex-adjusted)	1.31 [1.18; 1.46], *p*-value 5.44*10^−07*^	1.26 [1.12; 1.41], *p*-value 0.0001^*^	1.32 [1.14; 1.53], *p*-value 0.0003^*^	1.17 [0.97; 1.42], *p*-value 0.099	1.51 [1.19; 1.91], *p*-value 0.0006^*^
IHD (age-, sex-, and other factors-adjusted)	1.31 [1.18; 1.46], *p*-value 3.73*10^−07*^	1.27 [1.13; 1.43], *p*-value 5.24*10^−05*^	1.34 [1.16; 1.56], *p*-value 0.000136^*^	1.17 [0.97; 1.41], *p*-value 0.1215	1.47 [1.16; 1.86], *p*-value 0.0014^*^

**FIGURE 2:**
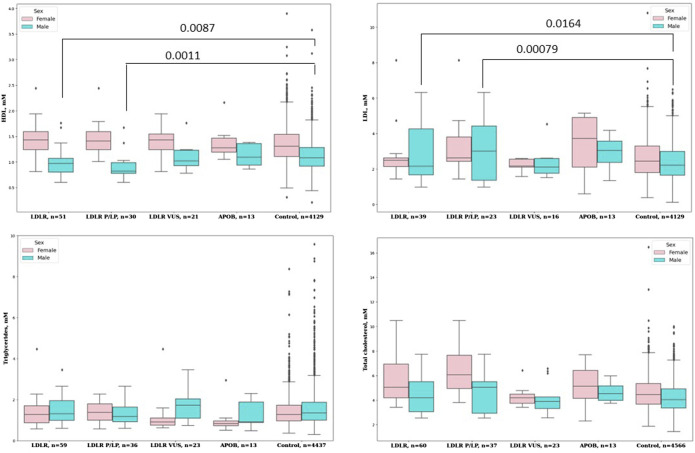
Lipid profiles of the male and female carriers of PVs, LPVs, or VUSs predicted to be deleterious.

Figure 2 shows the lipid profiles of the male and female carriers of PVs, LPVs, or VUSs predicted to be deleterious.

## Discussion

In this cohort study, we used whole-genome sequencing to examine genetic data from 4,856 Russian adult inpatients hospitalized with various CVDs. A total of 1.77% of inpatients carried *LDLR*, *APOB,* and *PCSK9* variants potentially associated with CVDs and an increased risk of IHD. The prevalence of PVs and LPVs was 3.3 times higher than that observed in the general adult Russian population (Meshkov AN. et al., 2021). The prevalence of PVs and LPVs in the *LDLR*, *APOB*, and *PCSK9* genes ranges from 0.4% in the general population to 4.4% in patients with myocardial infarction (Zhou et al., 2024; Ramensky et al., 2021; Cui et al., 2019). The examined cohort did not carry PVs or LPVs in the *PCSK9* gene. This finding is consistent with reports indicating that patients with FH are more likely to carry PVs or LPVs in the *LDLR* and *APOB* genes (Razman et al., 2022; Meshkov et al., 2021b). It also aligns with the data from the Russian register of patients with FH who underwent genetic testing (13% (n = 288)): PVs and/or LPVs in the *LDLR* gene were identified in 86% (n = 183) of patients, those in *APOB* were identified in 12% (n = 26), and those in *PCSK9* were identified in 2% (n = 4) (Chubykina et al., 2023).

Carriers of PVs and/or LPVs in the *LDLR* or *APOB* genes exhibited significant differences in the total cholesterol and LDL-C levels. However, after adjusting for statin use, the comparison of lipid profiles between carriers and non-carriers—stratified by sex— showed a significant difference only among men. Male carriers of PVs and/or LPVs presented with significantly higher levels of total cholesterol and LDL-C levels and lower HDL-C levels. However, these differences were not observed in carriers of *LDLR* variants interpreted as VUSs predicted to be deleterious by MetaLR and MetaSVM and carriers of PVs and/or LPVs in *APOB*. These findings indicate that *LDLR* variants may play a more prominent role in the development of FH. Meanwhile, the absence of significant associations with VUSs predicted to be deleterious may indicate either the low penetrance of these variants or an insufficient sample size.

Patients with PVs and/or LPVs in *LDLR* or *APOB* had a significantly higher risk of IHD (OR = 1.31; CI [1.18–1.46]; p-value = 5.44*10–07). Moreover, the prevalence of conduction and heart rhythm disorders was significantly higher in carriers of potentially deleterious variants, which may underlie the higher prevalence of IHD in this cohort (Kornej et al., 2020). These findings highlight the need for genetic screening of younger individuals for increased risk of IHD, thereby enabling early preventive and therapeutic interventions. Marquina et al. (2022) have demonstrated the economic benefit of this approach.

Underdiagnosed FH associated with PVs and/or LPVs presents an additional challenge. In our study, only 8 (15.7%) of the 51 patients with PVs and/or LPVs in the *LDLR* or *APOB* genes had a prior diagnosis of FH. However, based on the medical histories, excluding family histories and physical examination findings, 12 participants (23.5%) met the FH diagnostic criteria. However, it should be mentioned that FH diagnosis cannot rely solely on the detection of PVs and/or LPVs in the *LDLR*, *APOB*, and *PCSK9* genes due to the limited sensitivity of the FH diagnostic criteria—especially for younger individuals—and the incomplete penetrance of causal variants. In our cohort, penetrance, defined as LDL-C levels ≥ 4.0 mmol/L, ranged from 50% in the carriers of rare variants to 81% and 86.7% in *LDLR* and *APOB* variant carriers, respectively. Dikilitas et al. (2023) reported a penetrance of 87.5% in the carriers of PVs and/or LPVs in the *LDLR*, *APOB*, and *PCSK9* genes. These findings underscore the importance of implementing routine genetic screening for FH in younger individuals.

The study examined only predominantly Russian CVD inpatients. Therefore, its findings may not be applicable to a broader population or other ethnic groups. The study investigated the associations between genetic variants in the *LDLR* and *APOB* genes, lipid levels, and IHD. It did not consider other genes or environmental factors affecting lipid metabolism and the risk of cardiovascular diseases. Future studies involving larger, more ethnically and demographically diverse cohorts are needed to corroborate the findings of this study and further explore the interplay between genetic and environmental factors influencing lipid metabolism and cardiovascular health.

## Conclusion

Among 4,856 adult inpatients with CVDs, whose genetic data were examined through whole-genome sequencing, 1.77% were carriers of *LDLR* and/or *APOB* gene variants potentially associated with FH. No carriers of the *PCSK9* gene variants were identified in the examined cohort. The risk of IHD, adjusted for sex and age, was 1.31 times higher in the carriers of these variants [95% CI: 1.18–1.46; p = 5*10–7]. FH was found to be underdiagnosed, with only 10.5% of the *LDLR* and *APOB* gene variant carriers previously diagnosed with FH. This finding indicates the need for routine genetic screening of younger individuals for FH. However, further research is needed to evaluate the clinical applicability and cost-effectiveness of this approach.

## Data Availability

What is already known on this topic. Familial hypercholesterolemia is a common genetic dis- order associated with a higher risk of premature ischemic heart disease, and understanding its genetic prevalence could inform the feasibility of population-based genetic screening. What this study adds. This study identified that 1.77% of patients with cardiovascular diseases carry pathogenic or likely pathogenic variants in the LDLR and APOB genes, which are linked to a significantly higher risk of ischemic heart disease and elevated cholesterol levels. How this study might affect research, practice, or policy. The findings suggest that familial hypercholesterolemia is underdiagnosed, indicating the potential value of routine genetic screening in younger populations, although further research is necessary to evaluate the clinical and economic via- bility of such screening.

## Data availability statement

The data that support the findings of this study are available from the Centre for Strategic Planning of FMBA of Russia but restrictions apply to the availability of these data, which were used under license for the current study, and so are not publicly available. Data are however available from the authors upon reasonable request and with permission of the Centre for Strategic Planning of FMBA of Russia.

## Abbreviations

Potentially deleterious variants, genetic variants that increase an individual’s susceptibility or predisposition to a certain disease or disorder; selected variants, variants chosen for the initial or further analysis; PVs, pathogenic variants; LPVs, likely pathogenic variant; VUS, variant of uncertain significance.
